# Preparation and Characterization of Alginate and Psyllium Beads Containing *Lactobacillus acidophilus*

**DOI:** 10.1100/2012/680108

**Published:** 2012-05-02

**Authors:** Farzaneh Lotfipour, Shahla Mirzaeei, Maryam Maghsoodi

**Affiliations:** ^1^Faculty of Pharmacy, Tabriz University of Medical Sciences, Tabriz 51664, Iran; ^2^Gastrointestinal and Liver Disease Research Center, Tabriz University of Medical Sciences, Tabriz, Iran; ^3^Faculty of Pharmacy, Kermanshah University of Medical Sciences, Kermanshah, Iran; ^4^Biotechnology Research Center, Tabriz University of Medical Sciences, Tabriz, Iran

## Abstract

This paper describes preparation and characterization of beads of alginate and psyllium containing probiotic bacteria of *Lactobacillus acidophilus* DMSZ20079. Twelve different formulations containing alginate (ALG) and alginate-psyllium (ALG-PSL) were prepared using extrusion technique. The prepared beads were characterized in terms of size, morphology and surface properties, encapsulation efficiency, viabilities in acid (pH 1.8, 2 hours) and bile (0.5% w/v, 2 hours) conditions, and release in simulated colon pH conditions. The results showed that spherical beads with narrow size distribution ranging from 1.59 ± 0.04 to 1.67 ± 0.09 mm for ALG and from 1.61 ± 0.06 to 1.80 ± 0.07 mm for ALG-PSL with encapsulation efficiency higher than 98% were achieved. Furthermore, addition of PSL into ALG enhanced the integrity of prepared beads in comparison with ALG formulations. The results indicated that incorporation of PSL into alginate beads improved viability of the bacteria in acidic conditions as well as bile conditions. Also, stimulating effect of PSL on the probiotic bacteria was observed through 20-hour incubation in simulated colonic pH solution. According to our *in vitro* studies, PSL can be a suitable polymer candidate for partial substitution with ALG for probiotic coating.

## 1. Introduction

“Probiotics are live microorganisms (bacteria or yeasts), which when ingested or locally applied in sufficient numbers confer one or more specified demonstrated health benefits for the host” [1]. These benefits include maintenance of normal intestinal microflora, defense against enteropathogen infections, controlling serum cholesterol levels, improving lactose utilization in persons who are lactose maldigesters by production of *β*-galactosidase, and possessing anticarcinogenic and antimutagenic activities [2–4].

Probiotics can be bacteria, moulds, and yeasts. However, most of probiotics are bacteria; among them lactic acid bacteria (LAB), typically associated with the human gastrointestinal tract, are the most widely used probiotic microorganisms. In order to exhibit their potential benefits, probiotics need to pass the harsh conditions of gastric tract and colonize and grow on the epithelium of colon in appropriate population [5]. It is suggested that probiotics should be formulated in products in a minimum count of 10^6-7^ CFU/g or mL of viable probiotic bacteria [1]. To improve viability and stability of probiotics and efficient delivery of the cells to their active sites, various techniques have been utilized so far. In this regard, encapsulation of probiotics in wide variety of polymers is the most frequently applied method that is cited in numerous studies [6].

Alginate, a commonly used material to encapsulate probiotics, is a naturally occurring biocompatible and biodegradable linear anionic polysaccharide. Preparation of alginate bead, with well retained bacteria in their matrix, can be easily achieved by simple techniques like extrusion or emulsion methods. In spite of the wide application of alginate microcapsules in this area, some problems related to protection efficiency of them have been reported including susceptibility to disintegration in the presence of excess monovalent ions, Ca^2+^ chelating agents, and harsh chemical environments [4].

Psyllium, the common name used for several members of the plant genus *Plantago, *is gel-forming mucilage composed of a highly branched arabinoxylan. The backbone consists of xylose units, while arabinose and xylose form the side chains [7, 8]. Psyllium has been reported as a medicinally active natural polysaccharide for the treatment of constipation, diarrhea, irritable bowel syndrome, inflammatory bowel disease ulcerative colitis, colon cancer, diabetes, and hypercholesterolemia [9]. Moreover, psyllium as a soluble fiber has a potential to stimulate bacterial growth in digestive system, and, in some reports, it has been used as prebiotic [10–13]. Prebiotics is defined by Gibson and Roberfroid [14] as “non-digestible food ingredients that beneficially affects the host by selectively stimulating the growth and/or activity of one or a limited number of bacteria in the colon, and thus improves host health.”

Having in mind the pharmacological benefits of psyllium in digestive system as well as its potential to stimulate probiotic growth in the colon, here we aimed to incorporate psyllium in alginate beads containing probiotic bacteria *L. acidophilus *DMSZ20079. 

To this end, different formulations containing ALG and/or ALG-PSL were prepared using extrusion technique and characterized in terms of size, morphology and surface properties, encapsulation efficiency (EE), viabilities in acid (pH 1.8, 2 hours) and bile (0.5% w/v, 2 hours) conditions, and release in simulated colon pH conditions.

## 2. Materials and Methods

### 2.1. Materials

*L. acidophilus* DSMZ20079 was obtained from DSMZ (Germany), pepsin, pancreatin, sodium alginate, oxgall from Sigma-Aldrich (Germany), MRS broth and MRS agar, sodium hydrogen phosphate, calcium chloride, sodium hydroxide and hydrochloric acid from Merck (Germany), and psyllium seed husk was supplied from Sidpur Sat Isabgol (India).

### 2.2. Methods

#### 2.2.1. Preparation of Inoculum

*L. acidophilus* was cultured in MRS broth at 37°C for 18 hours. Culture was harvested by centrifugation at 700 RCF at 4°C for 7 min and washed twice with saline and collected by centrifugation as above. The washed bacterial cells were resuspended in 7 mL saline, and the cell count was determined using pour plate technique in MRS agar in triplicate. The cell suspension divided in some equal parts and consequently was used to prepare different formulations.

#### 2.2.2. Extraction of Psyllium

Psyllium husk was extracted by a method described by Guo et al. [8] with some modifications. First, 5 g psyllium husk was dispersed in 100 mL water at 80°C over night under constant stirring at 50 rpm, after 18–20 hours the dispersion became a homogenous gel. Consequently, the obtained gel was centrifuged (Hettich Rotofix 32 A, Germany) at 18000 RCF for 90 min, to separate the gel and the solution. The gel phase was dissolved in 2 M NaOH solution at room temperature for 2 hours; alkaline solution was separated from the residue by centrifugation (18000 RCF for 90 min) and accordingly neutralized with 2 M HCl. During the neutralization, a large amount of gel-like yellow precipitate was produced and separated by centrifugation (18000 RCF for 90 min) from the soluble fraction and washed three times with distilled water. The gel precipitate was dried at 40°C for 48 hours.

#### 2.2.3. Preparation of Beads

The extrusion technique was used to prepare ALG and ALG-PSL beads [15]. Sodium alginate and psyllium solutions were sterilized at 121°C for 15 min. The cooled ALG or ALG-PSL solutions (20 mL) were mixed with bacterial inoculum and gently stirred for 30 min to obtain a homogeneous suspension. The suspensions were extruded dropwise through a 27 gage nozzle into sterile hardening solution (CaCl_2_). The beads were shaken at 150 rpm for 40 min, isolated by aseptic filtration (Whatman No.1), washed twice with sterile water, and kept in 0.1% w/v peptone solution at 4°C. The prepared formulations are shown in Table 1.

#### 2.2.4. Size and Morphological Analysis

The particle size of beads was assessed using optical microscopy (Dino-lite, Taiwan) by Scion image analyzer software. Data were collected from 60 beads in each sample, and mean particle size was reported.

The topographical properties of prepared beads were investigated by scanning electron microscopy (SEM) (Philipse XL30, Holland) at an accelerating voltage of 20 KV. Prior to examination, samples were prepared on aluminum stubs and coated with gold under argon atmosphere by means of a sputter coater.

#### 2.2.5. Encapsulation Efficiency (EE)

To determine the encapsulation efficiency, firstly prepared beads were mechanically disintegrated in phosphate buffer (pH = 6.8), then the number of entrapped cells after adequate dilution were measured by pour plate method, and counts were expressed as number of colony forming units (CFU), and calculated as

(1) $$\text{EE}=\left(\frac{{\text{Log}}_{10}N}{{\text{Log}}_{10}N_{0}}\right)\times 100,$$

where *N* is the number of viable entrapped cells released from the beads and *N*_0_ is the number of free cells added to the biopolymer mixture immediately before the production procedure.

#### 2.2.6. Viability of Encapsulated and Free *L. acidophilus* at Low pH Condition

Low pH conditions were produced using 9 g/L sodium chloride and 3.0 g/L of pepsin and pH adjusted to 1.8 with hydrochloric acid [16]. 100 mg beads with entrapped bacteria or 0.1 mL of cell suspension were mixed in 20 mL of acid solution and incubated for 120 min at 37°C with constant agitation at 50 rpm. After incubation, beads were disintegrated in phosphate buffer (pH = 6.8), then 1.0 mL aliquot of the mixture removed and assayed using pour plate method.

The survival (%) of the bacteria was calculated as follows:

(2) $$\begin{array}{cc} & \%\;\text{Survival}\\ & =(\log\;\text{CFU}/\text{g}\;\text{beads}\;\text{after}\;2\;\text{hours}\;\text{exposure}\;\text{to}\;\\ & \;\text{acidic}\;\text{condition}/\log\;\text{CFU}/\text{g}\;\text{beads}\;\text{initial}\;\text{count})\times 100. \end{array}$$

#### 2.2.7. Viability of Encapsulated and Free *L. acidophilus* at High Bile Salt Concentration

Prepared beads after 2-hour acid exposure were washed with distilled water, removed, and incubated in 50 mL of high bile condition, containing 6.8 g of monobasic potassium phosphate, and 10 g/L of pancreatin with pH adjusted to 6.8 ± 0.1 using sodium hydroxide and 0.5% w/v oxgall for 2 hours at 37°C with constant agitation at 50 rpm [17]. Samples were then taken, and bacterial growth was assayed using pour plate method.

#### 2.2.8. Release of Encapsulated Cells and Free *L. acidophilus* in Simulated Colonic pH Solution

The release of the prepared beads was examined at simulated colonic pH solution as described by Mandal et al. [18]. The beads were mixed with 50 mL of simulated colonic pH solution containing 0.1 M monobasic potassium phosphate with pH adjusted to 7.4 ± 0.1 with sodium hydroxide and incubated for 20 h at 37°C with constant agitation at 50 rpm. Samples were taken at different time intervals, and bacterial growth was assayed using pour plate method as described in Section 2.2.5.

#### 2.2.9. Statistical Analyses

Statistical testing was carried out using SPSS19. All of the experiments were performed in triplicates. Data are presented as mean ± SD. The One-Way ANOVA test was performed to assess the difference between different beads and control groups and *P* < 0.05 considered as a statistically significant difference.

## 3. Results and Discussion

### 3.1. Characterization of Prepared Beads: Size, Morphology, Encapsulation Efficiency, and Surface Characteristic

In the preliminary experiments, different concentrations of ALG (0.75 to 3% w/v) and CaCl_2_ as hardening solution (1 to 6% w/v) were examined. According to the results of this step, it was found that uniform and spherical bead preparation by ALG concentrations less than 1% (w/v) was quite difficult because of decreased viscosity and less ion sites for cross-linkage [19]. Also, ALG concentrations more than 2% (w/v) were too viscose to be extruded from the syringe. Hence, the ALG concentrations between 1 to 2% w/v were selected. Moreover, according to our tests, 4% w/v CaCl_2_ produced the best result and chosen as optimum hardening solution.

In the second step of preliminary tests, the concentrations of PSL to be incorporated in ALG beads were optimized. Addition of PSL into ALG gel results in an increase in the viscosity and adherence of resultant gel. Indeed, incorporation of PSL in the concentrations more than 0.3, 0.5, and 0.6% w/v to ALG in the concentrations of 2, 1.5, and 1% w/v, respectively, yields too adherent mixtures to easily fabricate the beads. Consequently, the compositions in Table 1 were selected as the formulations to be further analyzed.

Table 2 shows results for diameters and encapsulation efficiencies of different ALG and ALG-PSL beads. As it can be seen, beads ranging from 1.59 to 1.67 mm for ALG and from 1.61 to 1.80 mm for ALG-PSL formulations were achieved. The mean diameters of beads containing PSL were significantly higher than those without PSL (*P* < 0.05) that can be attributed to the viscosity of the resultant gel. According to the studies in this regard, an increase in the viscosity of the starter gel leads to the preparation of bigger beads by the extrusion method [5]. Furthermore, narrow range of size distribution was observed for all prepared beads and no significant differences in size (*P* > 0.05) were observed between beads contained or not *L. acidophilus* loads. 

**Figure 1:**
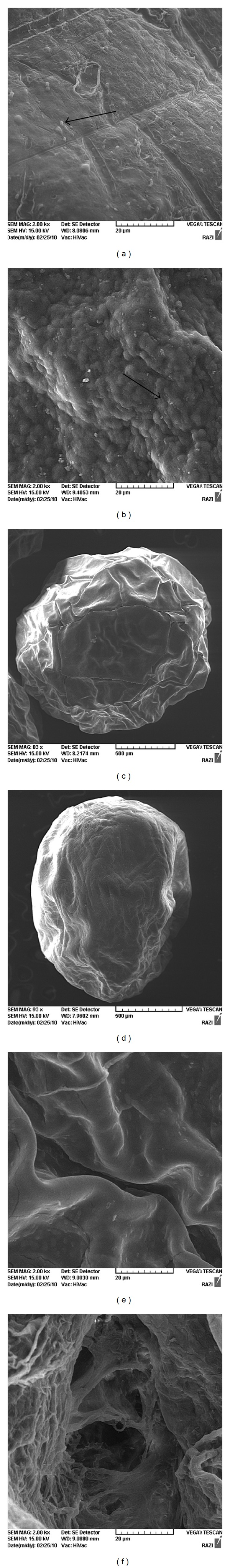
SEM pictures of F1 (a, c); F4 (b, d); F9 (e); F12 (f) beads at a magnification of 2000x (a, b, e, f), of 97x (c) and of 83x (d).

Scanning electron microscopy images of our formulations also show that the resultant beads are in spherical shape (Figures 1(c) and 1(d)) with groups of entrapped bacteria evident in the surfaces of the matrix (Figures 1(a) and 1(b)). The beads prepared using ALG concentrations of 2% w/v (F1) exhibited a smooth surface (Figure 1(a)) and relative small pores. On the other hand, optimum beads made using 1% w/v ALG have a rough surface and markedly open and large pores (Figure 1(e)), that is in good agreement with vast majority of other studies regarding the optimization of alginate concentration for probiotic microencapsulation [20]. On the other hand, as depicted in Figure 1(f), inclusion of PSL into ALG obviously lowered the surface roughness and pores of prepared beads and interestingly in the presence of PSL, even lower concentrations of ALG could also produce smoother surface with reduced pores (Figure 1(f)). 

The initial cell count of *L. acidophilus* before bead preparation was 9.81 ± 0.02 log CFU/mL. High bacterial cell entrapping in the range of 9.6 ± 0.06 to 9.78 ± 0.06 (log CFU/g beads) was achieved in resultant beads (Table 2). The results pertaining to EE indicated that there was no considerable loss of viability for all prepared beads and more than 98.9% cells for all beads were successfully entrapped that can be due to the gentle method applied [4]. Furthermore, no significant differences (*P* > 0.05) regarding the EE were observed between all formulations. 

### 3.2. Viability of Free and Encapsulated Bacteria in Acid Conditions

**Figure 2:**
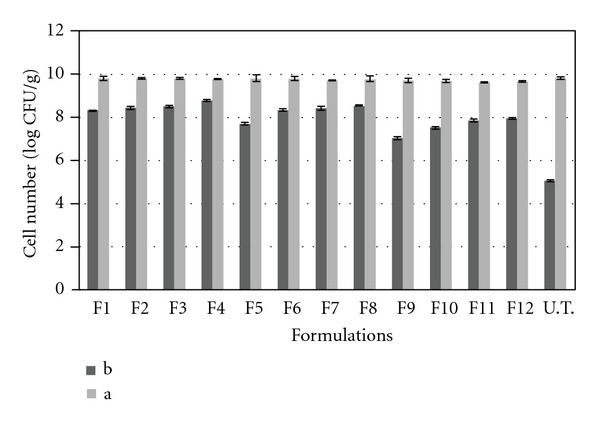
The viability of *L. acidophilus* (CFU/g) encapsulated in different ALG or ALG-PSL beads (F1–F12) and untreated cells. (a) Counts of the bacteria after 2 h acid exposure, (b) Initial counts of prepared beads and untreated cell count.

The protective effects of different coats of ALG and ALG-PSL after 2-hour exposure to acid conditions (pH = 1.8) are compared to untreated cells, and results are expressed as log CFU/g in Figure 2 and % survival in Table 2. 

As it can be seen from bar graphs in Figure 2, the initial inoculum count of untreated *L. acidophilus *was 9.81 ± 0.08 log CFU/g which declined to 5.06 ± 0.06 log CFU/g after acid exposure for 2 hours (around 39% survival). On the other hand, in our prepared beads with the initial cell numbers ranged between 9.6 ± 0.06 to 9.8 ± 0.03 log CFU/g, after 2 h acid exposure, the counts were 7.03 ± 0.1 to 8.43 ± 0.04 log CFU/g indicating more than 70% survival in all formulations. Overall, it is clear that survived bacteria after acid exposure, in all prepared beads were significantly (*P* < 0.05) higher than those of untreated cells. In fact, around 5 log reduction in bacterial count in the case of untreated *L. acidophilus *decreased to 1–3 log reduction among our obtained beads after 2 h acid exposure, and it can be concluded that coating of the bacteria as ALG or ALG-PSL beads can improve the viability of *L. acidophilus *in that conditions. There are numerous studies in this regard to protect probiotics by encapsulation in alginates beads using different techniques [21]. However, obtained results are controversial. In some cases, the investigations support our finding about the ability of ALG coat in protection of bacteria in acid conditions [15, 19, 22, 23]. For instance, Sohail et al. reported that encapsulation of probiotic bacteria in cross-linked alginate beads is of major interest for improving the survivability in harsh acid and bile environment [2]. Furthermore, Mokarram and collogues showed the efficiency of multistage alginate coating on survivability of probiotic bacteria in simulated gastric and intestinal juices [4]. However, Sultana and coworkers found that encapsulation of bacteria in alginate beads did not effectively protect the organisms from high acidity [24]. 

On the other hand, incorporation of PSL into alginate beads resulted in a rise in the viability of *L. acidophilus *in those beads in acid conditions and this effect is more obvious in higher concentrations of PSL. For instance, incorporation of 0.1 and 0.6% w/v PSL into 2% w/v (F2) and 1% w/v (F12) ALG solutions increased the survival around 1% and 12%, respectively. The increase in viability of the bacteria by addition of PSL is in line with our expectations, and it can be attributed to the total concentration of polymers blend used, as the survival of *L. acidophilus *in the beads with the same total amount of polymers showed no significant differences (*P* > 0.05) (F1 and F8 or F5 and F11). Moreover, the rising trend in the viability values by increase in the proportion of PSL can be attributed to the alginate concentration. As in the lower concentrations of alginate (1% w/v), the PSL effect is more dominant probably due to the fact that ALG concentration is insufficient for protection and addition of PSL increases the total polymer concentration and brings it to the appropriate point to remarkably increase the protection of the cells against acid conditions. Polymer blending is a simple yet attractive method to provide combined properties of polymers to a system and overcome their limitations [25]. This kind of compositions is widely used in encapsulation of probiotics. For instance, Albertini and coworkers, in good agreement with our results, reported that the incorporation of XG or CAP within the 3% w/v of alginate solution increased the survival of the probiotic bacteria in acid conditions [3]. Moreover, encapsulation of probiotic in alginate-starch blend [26] also showed improved level of protection against acidic condition. 

### 3.3. Viability of Encapsulated and Free *L. acidophilus* at High Bile Salt Concentration

**Figure 3:**
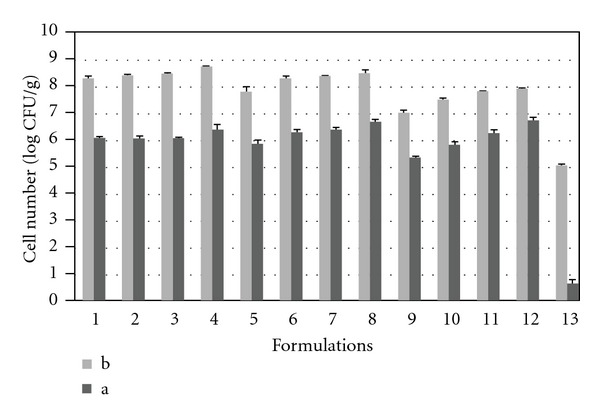
The viability of *L. acidophilus* (CFU/g) encapsulated in different ALG or ALG-PSL formulations (F1–F12) and untreated cells. (a) The counts after 2 h acid exposure, (b) The counts after 2 h acid exposure followed by 2 h bile exposure.

The effect of 2 h exposure to 0.5% w/v oxgall on the survival of *L. acidophilus *(passed through acidic conditions) in prepared beads and untreated cells is demonstrated in Figure 3. 

It is clear that *L. acidophilus* encapsulated in either ALG or ALG-PSL beads showed better survivability (less than 2.5 log reduction) after 2 h bile exposure compared to those of untreated (*P* < 0.05) that is dropped by around 4.5 log CFU/mL after 2 h bile exposure. According to the previous studies, survivability of encapsulated probiotics against harsh environmental conditions especially bile tolerance is highly dependent on the strain type. Our finding also concurs with the studies of Sohail et al., who reported that encapsulation of *L. acidophilus* in extruded macrobeads was effective in maintaining cell viability [2]. 

However, between the prepared beads, addition of less than 0.4% w/v PSL into ALG did not show significant changes in the viability of the bacteria in the presence of 0.5% w/v oxgall (*P* < 0.05) and the positive effect of PSL on the survivability of the bacteria is more evident in the lower concentrations of ALG. As a same manner with acid exposure results, we can discuss here that the rise in the total amount of polymer can be probably the reason of this phenomena. For instance, incorporation of 0.6% w/v PSL into 1% w/v ALG solution in F12 results in the fabrication of beads that were significantly more protective for the bacteria against bile condition compared to beads prepared just with 1% w/v ALG solution (F9). 

### 3.4. Release of Encapsulated and Free Bacteria in Simulated Colonic pH Solution

**Figure 4:**
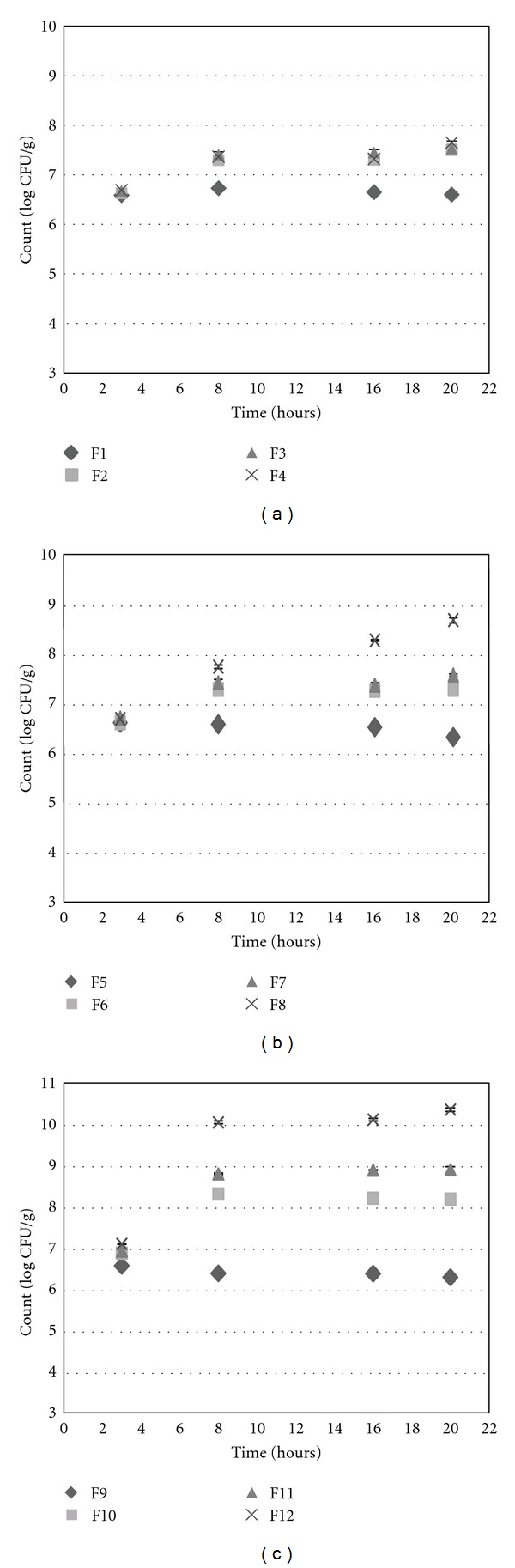
Release of *L. acidophilus* (CFU/g) in simulated colon pH solution (a) formulations F1–F4 and untreated cells (UT); (b) formulations F5–F8 and UT; (c) formulations F9–F12 and UT.

The bacterial release profile from the prepared beads in simulated colonic pH solution at different time intervals is illustrated in Figure 4. The release of probiotic bacteria from the beads and their colonization and growth in colon is crucial to take the advantages of the beneficial cells. Indeed if the prepared system does not disintegrate and release its payloads in adequate time, it leaves the body without producing any claimed benefits [18]. According to our results, during the first 3 h incubation in simulated colon pH solution, the cells increasingly released from all of the prepared beads to reach the level ranged 6-7 CFU/g, where the situation started to be remarkably different for coats of ALG-PSL in comparison with those of ALG. 

Among beads prepared with ALG (F1, F5, and F9), in 3 h, the counts hit their maximum level and beyond that no significant changes were observed in cell numbers (*P* > 0.05). It can be concluded that the ALG concentrations had no considerable effect on the release of *L. acidophilus* from the prepared beads. This finding also is in good agreement with Mandal et al. [18]. They reported that the count of *L. casei* in simulated colonic pH solution rose to its highest point in 60 min and after that remained constant. Moreover, according to Picot and Lacroix, a progressive release of coated cells in whey protein in simulated intestinal conditions occurred [27]. 

In sharp contrast, in the case of beads prepared using ALG-PSL, not only the bacteria completely released from the beads but also different rates of bacterial growth after 3 h was observed indicating a stimulating effect of PSL on the bacteria. Our data revealed that higher concentrations of PSL produced the greater stimulation effect on bacteria, as 0.6% w/v PSL in F12 showed more than 4 log rise in bacterial count. However, in F2 with minimum amount of PSL (0.1% w/v), lowest amount of growth was observed (under 2 log). The stimulation effect of PSL on *L. acidophilus* can be probably attributed to its structure as a soluble fiber. Based on prebiotic definition, nondigestible food ingredients such as carbohydrates in the form of soluble fiber can stimulate the growth and/or activity of bacteria. Indeed, in some cases, PSL has been used as prebiotic in different situations [10–13]. For instance, in a randomized controlled trial for treatment of patients with ulcerative colitis using synbiotic versus probiotic or prebiotic, PSL has been used as prebiotic and the results have shown that the quality of life in patients has been improved [10]. However, PSL has not been officially designated as a prebiotic so far and *in vivo* studies in this regards are in progress. In a study, the prebiotic potential of PSL fiber in healthy women on bifidobacteria was evaluated by Elli and colleagues [28], and they reported that PSL seed husk can be metabolized by bifidobacteria only after partial hydrolysis. 

In summary, whatever the prebiotic activity officially accepted or not for PSL, it is important to note that based on our data, it is assumable that PSL can potentially act as a prebiotic and preparation of PSL-ALG beads containing *L. acidophilus* improved its delivery to the active site. 

## 4. Conclusion 

ALG-PSL beads encapsulating probiotic *L. acidophilus *in the size range of 1.61 ± 0.06 to 1.80 ± 0.07 mm with EE higher than 98% were prepared using extrusion method. Inclusion of PSL into ALG beads maintained the survival rate of *L. acidophilus *in acid and bile conditions as well as considerable stimulation effect on the bacteria in simulated colon pH solution. Psyllium as a pharmacologically active ingredient for gastrointestinal disease and a potential prebiotic can be a suitable candidate to partially replace with alginate for encapsulation of probiotic bacteria. 

## Figures and Tables

**Table 1:** Compositions of the studied formulation.

Formulation	Alginate (% w/v)	Psyllium (% w/v)
F1	2	—
F2	2	0.1
F3	2	0.2
F4	2	0.3
F5	1.5	—
F6	1.5	0.3
F7	1.5	0.4
F8	1.5	0.5
F9	1	—
F10	1	0.4
F11	1	0.5
F12	1	0.6

**Table 2:** Size, encapsulation efficiency, and % survival in acid condition of prepared formulations.

Formulation	Diameter (mm) (*n* = 60)	Count (CFU/g) after preparation	Encapsulation efficiency (%)	% Survival
F1	1.67 ± 0.08	9.8 ± 0.03	99.8 ± 0.3	81.1 ± 1.1
F2	1.66 ± 0.04	9.79 ± 0.07	99.8 ± 0.9	82.3 ± 0.2
F3	1.65 ± 0.05	9.79 ± 0.06	99.7 ± 0.7	83.2 ± 0.7
F4	1.64 ± 0.03	9.76 ± 0.05	99.4 ± 0.6	86.6 ± 0.3
F5	1.64 ± 0.04	9.78 ± 0.06	99.7 ± 0.8	74.5 ± 2.5
F6	1.61 ± 0.06	9.78 ± 0.06	99.6 ± 0.7	80.9 ± 1.1
F7	1.65 ± 0.05	9.7 ± 0.09	98.6 ± 1.2	82.1 ± 0.4
F8	1.74 ± 0.05	9.75 ± 0.02	99.3 ± 0.3	83.4 ± 1.6
F9	1.59 ± 0.09	9.7 ± 0.07	98.6 ± 0.9	64.4 ± 1.5
F10	1.80 ± 0.07	9.6 ± 0.06	98.4 ± 0.8	70.7 ± 0. 7
F11	1.71 ± 0.04	9.61 ± 0.07	98.4 ± 0.8	74.7 ± 0.2
F12	1.77 ± 0.09	9.65 ± 0.04	98.0 ± 0.5	76.0 ± 0. 3
Untreated cells^a^	—	—	—	39.1 ± 0.8

^a^Inoculum count: 9.81 ± 0.08 CFU/mL.
